# Association between Neutrophil Levels on Admission and All-Cause Mortality in Geriatric Patients with Hip Fractures: A Prospective Cohort Study of 2,589 Patients

**DOI:** 10.1155/2022/1174521

**Published:** 2022-12-21

**Authors:** Rui Liu, Yan-Ning Zhang, Xu-Jing Fei, Jing-Ya Wang, Rong-Li Hua, Ying-Na Tong, Kun Li, Wen-Wen Cao, Shao-Hua Chen, Bin-Fei Zhang, Juan Chen, Yu-Min Zhang

**Affiliations:** Department of Joint Surgery, Honghui Hospital, Xi'an Jiaotong University, Beilin District, Xi'an, Shaanxi, China

## Abstract

**Objective:**

To evaluate the association between neutrophil levels and all-cause mortality in geriatric hip fractures.

**Methods:**

Elderly patients with hip fractures were screened between January 2015 and September 2019. Demographic and clinical characteristics of the patients were collected. Linear and nonlinear multivariate Cox regression models were used to identify the association between neutrophil levels and mortality. Analyses were performed using Empower Stats and R software.

**Results:**

A total of 2,589 patients were included in this study. The mean follow-up period was 38.95 months. During the study period, 875 (33.80%) patients died due to various causes. Linear multivariate Cox regression models showed that neutrophil levels were associated with mortality after adjusting for confounding factors, when neutrophil concentration increased by 1*∗*10^9^/L, the mortality risk increased by 3% (HR = 1.03, 95% CI: 1.00–1.06, and *P*=0210). Neutrophil concentration was used as a categorical variable; we only found statistically significant differences when neutrophil levels were high (HR = 1.27, 95% CI:1.05–1.52, and *P*=0.0122). In addition, the results are stable in *P* for trend and propensity score matching sensitivity analysis.

**Conclusions:**

Neutrophil levels are associated with mortality in geriatric hip fractures and could be considered a predictor of death risk in the long-term. This study is registered with the Chinese Clinical Trial Registry (ChiCTR) as number ChiCTR2200057323.

## 1. Introduction

Hip fractures are an important public health problem of global concern [1, 2]. With the advance of global aging and longer life expectancy, the incidence of geriatric hip fractures in many countries continues to rise dramatically [1, 3]. A systematic review of 63 countries showed that the rates (/100,000) of hip fracture in women were over 500 in four countries such as Denmark (574), Norway (563), Sweden (539), and Austria (501), with 25 regions having hip fracture rates higher than 300, and 21 regions having rates over 200 [4]. A previous study has estimated that hip fracture cases in China will increase six-fold, from 0.7 million cases in 2013 to 4.5 million cases in 2050 [5]. The main finding from China's seventh national population census was that an aging tsunami was coming [6]. In Japan, the lifetime risk of hip fracture in individuals aged 50 years was shown to be 5.6% for men and 20% for women [7]. Despite improvements in the treatment of geriatric patients with hip fractures, the mortality rate remains excessively high (25%–30% within 1 year and up to 40% within 3 years) [8–10]. In addition, 1.75 million disability-adjusted life years have been lost by hip fractures, representing 0.1% of the global disease burden [11]. Therefore, in the face of the increasing medical and health resources spent on hip fractures in the elderly, related research studies are highly necessary.

In worldwide practice, surgery is considered the treatment of choice for the majority of patients with hip fractures (>90%) [12–14]. At the same time, nonoperative management is associated with high mortality and complication rates, and recovery to pretrauma functioning is low [12]. Malnutrition was associated with an increase in mortality [15], and low scores in anthropometric indices (such as body mass index (BMI), weight loss, or albumin concentration) were associated with a higher prevalence of complications during hospitalization and worse functional recovery [8].

Neutrophils have traditionally been considered the simple frontline troops of the innate immune system, equipped with limited proinflammatory duties. In contrast, it is now known that neutrophils are complex cells capable of a significant array of specialized functions, and as effectors of the innate immune response, they exert a role in different processes such as acute injury and repair, cancer, autoimmunity, and chronic inflammation [16, 17]. In tissue injury, neutrophils contribute by amplifying the inflammatory response and directly releasing toxic effectors in order to restore tissue architecture and function [18–21]. According to some studies, the neutrophil-to-lymphocyte ratio (NLR) is positively associated with the severity of trauma [22] and is appropriate for monitoring mortality in patients with geriatric hip fracture [23–25].

However, the relationship between serum neutrophil levels and the prognosis of patients with hip fractures remains unclear. Therefore, this prospective cohort study aimed to assess the association of serum neutrophil levels with mortality in patients with hip fracture over a long-term follow-up period. We hypothesized that neutrophil levels and mortality would show either a linear or a nonlinear association.

## 2. Materials and Methods

### 2.1. Study Design

We recruited elderly patients who experienced a hip fracture between January 1, 2015, and September 30, 2019, at Northwest China's largest trauma facility.

This prospective study was approved by the Ethics Committee of the Xi'an Honghui Hospital (No. 202201009). All procedures involving human participants were performed in accordance with the 1964 Declaration of Helsinki and its amendments. The informed consent was oral by telephone.

### 2.2. Participants

Demographic and clinical data about the patients were obtained from their original medical records. The inclusion criteria were as follows: (1) those aged ≥65 years; (2) those with a radiographic or computed tomography diagnosis of a femoral neck, intertrochanteric, or subtrochanteric fracture; (3) patients who were receiving surgical or conservative treatment in a hospital; (4) availability of clinical data in the hospital; and (5) patients who could be contacted by telephone. Patients who could not be contacted were excluded from this study.

### 2.3. Hospital Treatment

The patient's blood samples were collected on admission and after the operation or discharge. Patients were examined using blood tests taken as presurgical testing. Intertrochanteric fractures were generally managed with closed or open reduction and internal fixation (ORIF) using a proximal femoral nail antirotation implant. Femoral neck fractures were generally treated with hemiarthroplasty (HA) or total hip arthroplasty (THA), depending on the patient's age. Prophylaxis for deep vein thrombosis was initiated on admission. Patients were asked to return monthly after discharge to assess fracture union or function.

### 2.4. Follow-Up

After discharge, the patients' family members were contacted by telephone from January to March 2022 to record data on survival, survival time, and activities of daily living. This follow-up was conducted by two medical professionals with two weeks of training and one year of experience. Patients who could not be contacted initially were referred two additional times. Whenever the family members of the patients did not respond, the recording and follow-up for the patient were stopped.

### 2.5. Endpoint Events

The endpoint event in this study was all-cause mortality. We defined all-cause mortality as deaths reported by the patients' family members.

### 2.6. Variables

The variables in our study were as follows: age, sex, occupation, history of allergy, injury mechanism, fracture classification, presence of hypertension, diabetes, coronary heart disease (CHD), arrhythmia, hemorrhagic stroke, ischemic stroke, cancer, multiple injuries, dementia, chronic obstructive pulmonary disease (COPD), hepatitis and gastritis, age-adjusted Charlson comorbidity index (aCCI), time from injury to admission, time from admission to operation, neutrophil count, treatment strategy, operation time, blood loss, infusion, transfusion, length of hospital, and follow-up.

Neutrophil levels were measured on admission. The dependent variable was all-cause mortality, while the independent variable was the neutrophil level. The other variables were potentially confounding factors.

### 2.7. Statistical Analysis

Continuous variables were reported as mean ± standard deviation (Gaussian distribution) or median (range and skewed distribution). Categorical variables are presented as numbers with proportions. Chi-square (categorical variables), one-way analysis of variance (ANOVA (normal distribution)), or the Kruskal–Wallis H test (for skewed distribution) were used to detect differences between different neutrophil levels. Univariate and multivariate Cox proportional hazards regression models (three models) were used to test the association between neutrophil levels and mortality. The nonadjusted model was not adjusted for covariates. Model I was a minimally adjusted model with adjusted sociodemographic variables. Model II was fully adjusted for all covariates. To test the robustness of our results, we performed a sensitivity analysis. We converted the neutrophil level into a categorical variable, calculated the *P* for trend to verify the results of neutrophils as a continuous variable, and examined the possibility of nonlinearity. Because Cox proportional hazards regression model-based methods are often suspected to be unable to deal with nonlinear models, the nonlinearity between neutrophils and mortality was addressed by adding cubic spline functions and smooth curve fitting (penalized spline method) to the Cox proportional hazards regression model. If nonlinearity was detected, we first calculated the inflection point using a recursive algorithm and then constructed a two-piecewise Cox proportional hazards regression model on both sides of the inflection point. In addition, propensity score matching (PSM) was used for comparison between matched groups, and we adjusted for confounding factors in the PSM models.

All analyses were performed using the statistical software packages R (http://www.R-project.org, R Foundation) and Empower Stats (http://www.empowerstats.com, X&Y Solutions Inc., Boston, MA, USA). Hazard ratios (HRs) with 95% confidence intervals (CIs) were calculated. Statistical significance was set at *P* < 0.05 (two sided) and was considered statistically significant.

## 3. Results

### 3.1. Patient Characteristics

**Figure 1:**
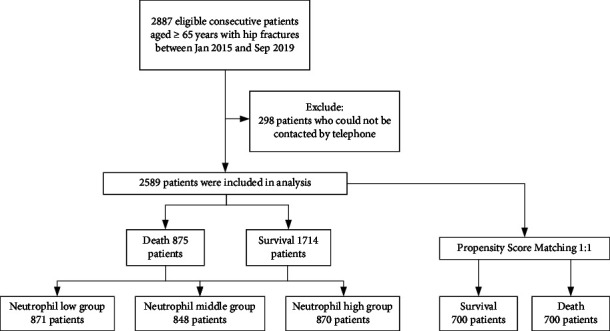
Study flow diagram.

From the initial 2,887 participants who had hip fractures between January 2015 and September 2019, 2,589 met the study criteria and were enrolled in our study. The mean follow-up was 38.95 ± 19.67 months. Two hundred ninety-eight patients were excluded because of a lack of follow-up. A total of 875 (33.80%) patients died due to all-cause mortality. We assessed the neutrophil levels in these patients and divided them into three groups according to their neutrophil levels (low, middle, and high). The flow chart is shown in Figure 1.

Table 1 lists the demographic and clinical characteristics of all 2589 patients including comorbidities, factors associated with injuries, and treatment. There were significant differences in these clinical parameters between the three groups, namely fracture classification, hypertension, cancer, hepatitis, treatment strategy, time to admission, operation time, and infusion.

### 3.2. Univariate Analysis of Association between Variates and Mortality

To identify potential confounding factors and the relationship between these variables and mortality, we performed a univariate analysis (Table 2). According to the criteria of *P*  <  0.1, the following variables were considered in the multivariate Cox regression: age, sex, injury mechanism, fracture classification, aCCI, hypertension, CHD, arrhythmia, ischemic stroke, cancer, dementia, COPD, hepatitis, time to admission and operation, treatment strategy, operation time, infusion, and neutrophil count.

### 3.3. Multivariate Analysis between Neutrophil and Mortality

We used three models (Table 3) to correlate neutrophil levels with mortality. When neutrophil level was a continuous variable, linear regression was observed. The fully adjusted model (Model II) showed a mortality risk increase of 3% (HR = 1.03, 95% CI: 1.00–1.06, *P*=0.0210) when the neutrophil concentration increased by 1*∗*10^9^/L after controlling for confounding factors. When neutrophil concentration was used as a categorical variable, we found statistically significant differences between the high and low neutrophil level groups. This instability indicates the possibility of a nonlinear correlation.

However, the *P* for trend also showed a linear correlation in these three models (*P* < 0.0001).

### 3.4. Curve Fitting and Analysis of Inflection Point

**Figure 2:**
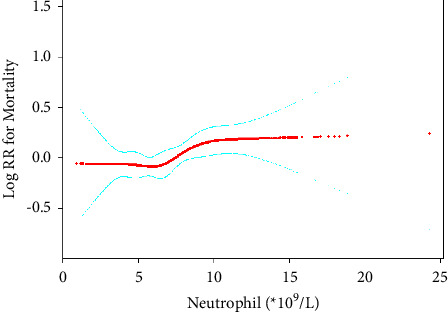
Curve fitting between neutrophil and mortality. Adjusted for age, sex, injury mechanism, fracture classification, aCCI, hypertension, CHD, arrhythmia, ischemic stroke, cancer, dementia, COPD, hepatitis, time to admission and operation, treatment strategy, operation time, and infusion.

As shown in Figure 2, there was a curved association between neutrophil count and mortality after adjusting for confounding factors. We compared two fitting models to explain this association (Table 4). Unfortunately, we did not observe an inflection point for the saturation or threshold effect.

### 3.5. PSM Sensitivity Analysis

**Figure 3:**
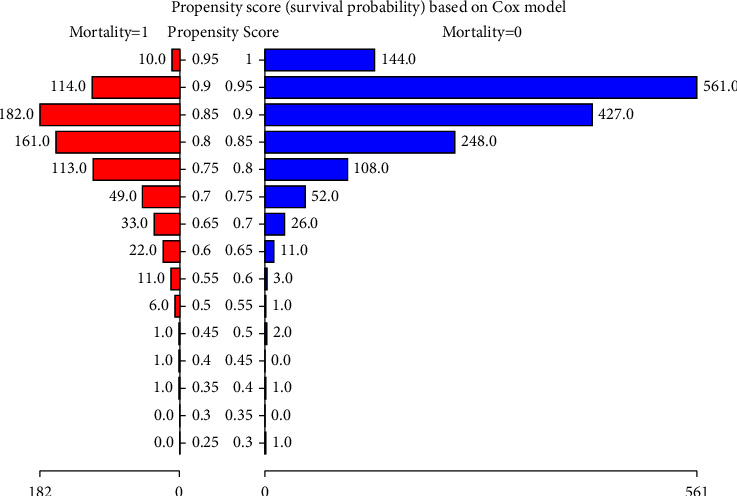
The PSM of two groups under propensity score based on Cox model.

To test the robustness of our results, we performed sensitivity analysis using PSM, as shown in Figure 3 and Tables 5–7. A total of 1,400 patients were successfully matched (Figure 3; Table 5). Age and aCCI did not match between the two groups (Table 6). In the multivariate Cox regression results under the PSM and PSM-adjusted models, the results were stable (Table 7).

## 4. Discussion

We found that there was a linear association between neutrophil levels and all-cause mortality in geriatric hip fractures, higher neutrophil levels were associated with higher mortality (HR = 1.03, 95% CI: 1.00–1.06; *P* = 0.0210). This finding indicated that for every 110^9/L additional neutrophils, the fatality rate increased by 3%. Compared with the low group, the fatality rate did not increase in the middle group (HR = 1.05, 95% CI: 0.87–1.27; *P*=0.5914); however, it was significantly higher in the high group (HR = 1.27, 95% CI: 1.05–1.52; *P*=0.0122). In addition, the results are stable in *P* for trend and PSM sensitivity analysis. Neutrophil levels can be considered a predictor of the risk of mortality in geriatric hip fractures in clinical practice.

Neutrophils are generally regarded as being beneficial to the host during infection, as neutropenic patients are at a high risk of infection-related mortality [19]. Human neutrophils have been shown to contribute to bone regeneration by rapidly infiltrating the hematoma associated with bone fractures and synthesizing fibronectin extracellular matrix within 48 h after injury (before stromal cells are present) [26]. However, locally increased neutrophil numbers have been shown to impair bone repair after severe trauma [27]. Interestingly, neutrophil depletion also impairs the fracture healing outcome [28]; therefore, an optimal neutrophil number is important for successful bone repair. To date, there are insufficient studies on the association of neutrophils with hip fracture prognosis, and most studies have rather focused on the relationship between hip fracture prognosis and the ratio of neutrophils to lymphocytes. A prospective study indicated that age, sex, and the ratio of neutrophils to lymphocytes were predictors of mortality in elderly patients during the first postoperative year following surgery for hip fracture [23]. In addition, Temiz's retrospective case-control study showed that the admission ratio of neutrophil and lymphocyte values of patients in the dead group was significantly higher than that of patients in the control group [25]. However, the effect of neutrophil levels on hip fracture fatality has not been explored separately.

In addition to the linear relationship, we speculated on a curvilinear relationship through subgroup analysis and curve fitting. However, we did not find an inflection point on the curve in this study. For this reason, the linear relationship is more appropriate to explain the relationship between neutrophil levels and geriatric hip fracture mortality. Similar to Fisher's findings, the ratio of neutrophil and lymphocyte values before surgery shows a linear correlation with geriatric hip fracture prognosis [29].

Neutrophils play an important role in bone homeostasis by expressing and secreting inflammatory mediators that can directly or indirectly affect mesenchymal stem cells, osteoblasts, and osteoclasts [30]. Neutrophils are part of the innate immune system, the first line of defense against microbial pathogens [31], and affect their functions, including aging [32]. Immunosenescence upon aging may be a major contributor to a decline in immune functions in both innate and adaptive immune systems, leading to an increased susceptibility to opportunistic infections [33–35]. In fact, neutrophils from healthy elderly individuals display reduced chemotactic and phagocytic activities [36, 37], whereas neutrophils from hip fracture patients have higher chemotactic and phagocytic activities [38]. Most older people who fracture a hip are frail, have comorbidities, and show functional deterioration typical of geriatric patients [39]. After a fracture, both short-term and long-term outcomes for patients are generally poor [40]. Therefore, age may be an important factor in the relationship between neutrophils and the risk of death in geriatric hip fractures, and the neutrophil level may likely have a predicting value.

C-reaction protein [41], interleukin-6 [42], and tumor necrosis factor-*α* [42] are all inflammation biomarkers that have been shown to predict mortality events in hip fracture patients. Norring–Agerskov et al. reported that an elevated level of C-reaction protein was associated with 30-day mortality after a hip fracture [41]. Bermejo–Bescos et al. reported that IL-6 was associated with a higher risk of 1-year mortality, but not tumor necrosis factor-*α* [42]. On the one hand, the neutrophil level was more convenient than these biomarkers of inflammation because the surgeon would not have a specialized serological test for these inflammatory indicators. On the other hand, the association between neutrophils and mortality was long-lasting. Our study showed that the neutrophil level was associated with long-term mortality in 38.95 months of follow-up. Thus, we believed that the neutrophil level was more suitable for predicting mortality. In clinical practice, we suggest the neutrophil level as a usual predictor of the long-term risk of death and consider neutrophils as an essential candidate in the predictive models and nomograms for elderly hip fractures.

In order to obtain a reliable conclusion, we identified possible confounding factors as well as neutrophil levels. As reported in previous studies, age, sex, fracture type, comorbidities, coronary heart disease, arrhythmia, tumor, dementia, time from injury to surgery, and treatment strategy are risk prognostic factors for hip fracture [12–14, 30, 43]. In addition, in the univariate analysis, we also found some factors with *P* < 0.1, including injury mechanism, ischemic stroke, operation time, and infusion volume. Considering the factors affecting neutrophil levels, we also included others such as COPD [44, 45] and tumor [46, 47]. Therefore, we controlled for a vast majority of confounding factors.

This study has some limitations. First, follow-up loss is unavoidable in a prospective cohort study, and this study is no exception. Therefore, we called several other contact numbers to reach those patients who could not be contacted initially in order to obtain their outcomes. Second, this study was unable to determine the causal relationship between neutrophils and prognosis, which should be confirmed in future studies. Third, our study population was derived from Western China; therefore, the conclusions have geographical and ethnic limitations. Fourth, in this study, we only focused on the neutrophil level on admission, not the dynamic changes of neutrophils. Therefore, this conclusion should be used cautiously by people in other regions.

In summary, neutrophil level was associated with mortality in geriatric hip fractures and could be considered a predictor of the risk of death in the long-term.

## Figures and Tables

**Table 1:** The demographic and clinical characteristics (*N* = 2589).

Neutrophil tertiles (^*∗*^10^9^/L)	Low group (0.92–5.43)	Middle group (5.44–7.71)	High group (7.72–24.86)	*P* value	*P* value^∗^
*N*	871	848	870		
Age (years)	79.43 ± 6.86	79.72 ± 6.88	79.61 ± 6.65	0.675	0.511
Sex				0.727	—
Male	278 (31.92%)	286 (33.73%)	285 (32.76%)		
Female	593 (68.08%)	562 (66.27%)	585 (67.24%)		
Neutrophil (^*∗*^10^9^/L)	4.22 ± 0.89	6.54 ± 0.65	10.00 ± 2.26	<0.001	<0.001
Occupation				0.611	—
Retirement	485 (55.68%)	488 (57.55%)	516 (59.31%)		
Farmer	222 (25.49%)	209 (24.65%)	198 (22.76%)		
Other	164 (18.83%)	151 (17.81%)	156 (17.93%)		
History of allergy	39 (4.48%)	31 (3.66%)	31 (3.56%)	0.556	—
Injury mechanism				0.27	—
Falling	840 (96.44%)	818 (96.46%)	843 (96.90%)		
Accident	20 (2.30%)	26 (3.07%)	22 (2.53%)		
Other	11 (1.26%)	4 (0.47%)	5 (0.57%)		
Fracture classification				<0.001	—
Intertrochanteric fracture	585 (67.16%)	627 (73.94%)	675 (77.59%)		
Femoral neck fracture	263 (30.20%)	203 (23.94%)	169 (19.43%)		
Subtrochanteric fracture	23 (2.64%)	18 (2.12%)	26 (2.99%)		
Hypertension	377 (43.28%)	425 (50.12%)	456 (52.41%)	<0.001	—
Diabetes	160 (18.37%)	171 (20.17%)	185 (21.26%)	0.312	—
CHD	445 (51.09%)	441 (52.00%)	491 (56.44%)	0.058	—
Arrhythmia	301 (34.56%)	273 (32.19%)	298 (34.25%)	0.531	—
Hemorrhagic stroke	26 (2.99%)	15 (1.77%)	16 (1.84%)	0.153	—
Ischemic stroke	241 (27.67%)	236 (27.83%)	278 (31.95%)	0.084	—
Cancer	36 (4.13%)	20 (2.36%)	19 (2.18%)	0.028	—
Multiple injuries	58 (6.66%)	58 (6.84%)	65 (7.47%)	0.784	—
Dementia	28 (3.21%)	43 (5.07%)	32 (3.68%)	0.123	—
COPD	60 (6.89%)	47 (5.54%)	61 (7.01%)	0.392	—
Hepatitis	41 (4.71%)	20 (2.36%)	20 (2.30%)	0.005	—
Gastritis	20 (2.30%)	15 (1.77%)	11 (1.26%)	0.265	—
aCCI	4.18 ± 1.06	4.21 ± 1.08	4.27 ± 1.10	0.234	0.169
Treatment strategy				<0.001	—
Conservation	61 (7.00%)	74 (8.73%)	90 (10.34%)		
ORIF	542 (62.23%)	583 (68.75%)	618 (71.03%)		
HA	250 (28.70%)	182 (21.46%)	154 (17.70%)		
THA	18 (2.07%)	9 (1.06%)	8 (0.92%)		
Time to admission (h)	140.31 ± 378.47	57.58 ± 144.38	44.13 ± 119.43	<0.001	<0.001
Time to operation (d)	4.11 ± 2.46	4.46 ± 2.73	4.35 ± 2.53	0.024	0.019
Operation time (mins)	91.16 ± 34.44	92.97 ± 38.64	98.21 ± 38.05	<0.001	<0.001
Blood loss (mL)	248.18 ± 164.28	254.20 ± 181.75	234.18 ± 138.97	0.05	0.099
Transfusion (U)	1.15 ± 1.25	1.21 ± 1.32	1.11 ± 1.25	0.327	0.413
Infusion (mL)	1545.87 ± 376.84	1540.57 ± 387.42	1593.84 ± 398.42	0.013	0.024
Follow-up (m)	39.03 ± 19.17	39.71 ± 19.93	38.12 ± 19.88	0.242	0.225
Mortality	273 (31.34%)	284 (33.49%)	318 (36.55%)	0.07	—

Mean + SD/*N* (%)*. P* value^∗^: for continuous variables, we used the Kruskal–Wallis rank sum test and Fisher's exact probability test for count variables with a theoretical number <10.

**Table 2:** Effects of factors on mortality measured by univariate analysis (*N* = 2589).

	Statistics	HR (95% CI)	*P* value
Age (years)	79.59 ± 6.80	1.08 (1.06, 1.09)	<0.0001
Sex			
Male	849 (32.79%)	1	
Female	1740 (67.21%)	0.74 (0.65, 0.85)	<0.0001
Occupation			
Retirement	1489 (57.51%)	1	
Farmer	629 (24.30%)	0.91 (0.78, 1.07)	0.2607
Other	471 (18.19%)	0.84 (0.70, 1.01)	0.0669
History of allergy	101 (3.90%)	0.88 (0.61, 1.27)	0.4854
Injury mechanism			
Falling	2501 (96.60%)	1	
Accident	68 (2.63%)	0.25 (0.12, 0.54)	0.0003
Other	20 (0.77%)	1.61 (0.86, 2.99)	0.1369
Fracture classification			
Intertrochanteric fracture	1887 (72.89%)	1	
Femoral neck fracture	635 (24.53%)	0.85 (0.71, 1.01)	0.0676
Subtrochanteric fracture	67 (2.59%)	0.78 (0.50, 1.20)	0.2584
aCCI	4.22 ± 1.08	1.51 (1.43, 1.61)	<0.0001
Hypertension	1258 (48.59%)	1.13 (0.99, 1.29)	0.0643
Diabetes	516 (19.93%)	1.01 (0.86, 1.20)	0.882
CHD	1377 (53.19%)	1.32 (1.15, 1.51)	<0.0001
Arrhythmia	872 (33.68%)	1.32 (1.15, 1.51)	<0.0001
Hemorrhagic stroke	57 (2.20%)	1.14 (0.74, 1.76)	0.5501
Ischemic stroke	755 (29.16%)	1.42 (1.24, 1.64)	<0.0001
Cancer	75 (2.90%)	1.77 (1.28, 2.44)	0.0005
Multiple injuries	181 (6.99%)	0.99 (0.76, 1.28)	0.9177
Dementia	103 (3.98%)	2.62 (2.03, 3.38)	<0.0001
COPD	168 (6.49%)	1.55 (1.23, 1.95)	0.0002
Hepatitis	81 (3.13%)	1.62 (1.17, 2.23)	0.0033
Gastritis	46 (1.78%)	0.95 (0.58, 1.57)	0.8533
Time to admission (h)	80.89 ± 248.16	1.00 (1.00, 1.00)	0.0531
Time to operation (d)	4.30 ± 2.58	1.03 (1.00, 1.05)	0.0481
Treatment strategy			
Conservation	225 (8.69%)	1	
ORIF	1743 (67.32%)	0.31 (0.26, 0.38)	<0.0001
HA	586 (22.63%)	0.33 (0.26, 0.41)	<0.0001
THA	35 (1.35%)	0.06 (0.02, 0.26)	0.0001
Operation time (mins)	94.08 ± 37.16	1.00 (1.00, 1.00)	0.0433
Blood loss (mL)	245.52 ± 162.69	1.00 (1.00, 1.00)	0.4246
Transfusion (U)	1.16 ± 1.27	1.05 (0.99, 1.11)	0.1136
Infusion (mL)	1559.80 ± 388.06	1.00 (1.00, 1.00)	0.0001
Neutrophil (^*∗*^10^9^/L)	6.92 ± 2.79	1.03 (1.00, 1.05)	0.0351

**Table 3:** Univariate and multivariate results by Cox regression (*N* = 2589).

Exposure	Nonadjusted	Model I	Model II
Neutrophil (^*∗*^10^9^/L)	1.03 (1.00, 1.05) 0.0351	1.03 (1.00, 1.05) 0.0315	1.03 (1.00, 1.06) 0.0210
Neutrophil tertiles			
Low (0.92–5.43)	1	1	1
Middle (5.44–7.71)	1.05 (0.89, 1.24) 0.5465	1.05 (0.89, 1.24) 0.5807	1.05 (0.87, 1.27) 0.5914
High (7.72–24.86)	1.19 (1.01, 1.40) 0.0343	1.21 (1.03, 1.42) 0.0236	1.27 (1.05, 1.52) 0.0122
*P* for trend	0.0334	0.0228	0.0119

Data in table: HR (95%CI) *P* value. Outcome variable: mortality. Exposed variables: neutrophil. Model I adjusted for: age and sex. Model II adjusted for age, sex, injury mechanism, fracture classification, aCCI, hypertension, CHD, arrhythmia, ischemic stroke, cancer, dementia, COPD, hepatitis, time to admission and surgery, treatment strategy, operation time, and infusion.

**Table 4:** Nonlinearity of neutrophil (^*∗*^10^9^/L) versus mortality (*N* = 2589).

Outcome	HR (95%CI) *P* value
Fitting model by stand linear regression	1.03 (1.00, 1.06) 0.0210
Fitting model by two-piecewise linear regression	
Inflection point	5.27
<5.27	0.99 (0.88, 1.11) 0.8471
>5.27	1.04 (1.01, 1.07) 0.0193
*P* for log-likelihood ratio test	0.462

Adjusted for age, sex, injury mechanism, fracture classification, aCCI, hypertension, CHD, arrhythmia, ischemic stroke, cancer, dementia, COPD, hepatitis, time to admission and operation, treatment strategy, operation time, and infusion.

**Table 5:** Propensity score parameter list.

The variables used in calculating the propensity score	Age, sex, injury mechanism, fracture classification, aCCI, hypertension, CHD, arrhythmia, ischemic stroke, cancer, dementia, COPD, hepatitis, time to admission and operation, treatment strategy, operation time, and infusion
Propensity score algorithm	Cox regression model
*C*-statistical	0.7044
Matching method	Greedy matching within specified caliper distances
Metric distances	0.05
Matching ratio	1 : 1
Use of replacement	With replacement

Matching sample size	No. of death = 1 : 700 cases
	No. of alive = 1 : 700 cases
	Total 1400 cases

**Table 6:** The balance test of PSM (*N* = 1400).

Variables	Mortality: alive (*N* = 700)	Mortality: dead (*N* = 700)	Standardized diff.	*P* value
Age (years)	83.33 ± 4.65	81.89 ± 6.39	0.258	<0.0001^∗^
Sex			0.0356	0.5419
Male	251 (35.9)	263 (37.6)		
Female	449 (64.1)	437 (62.4)		
Hypertension			0.04	0.487
No	335 (47.9)	349 (49.9)		
Yes	365 (52.1)	351 (50.1)		
CHD			0.0058	0.9571
No	310 (44.3)	312 (44.6)		
Yes	390 (55.7)	388 (55.4)		
aCCI				<0.0001^∗^
2	0 (0)	8 (1.1)	0.1521	
3	23 (3.3)	78 (11.1)	0.3073	
4	316 (45.1)	301 (43)	0.0432	
5	252 (36)	204 (29.1)	0.1467	
6	89 (12.7)	81 (11.6)	0.035	
7	18 (2.6)	22 (3.1)	0.0343	
8	2 (0.3)	6 (0.9)	0.0759	
Arrhythmia			0.0298	0.6157
No	455 (65)	445 (63.6)		
Yes	245 (35)	255 (36.4)		
Ischemic stroke			0.0031	1
No	473 (67.6)	474 (67.7)		
Yes	227 (32.4)	226 (32.3)		
Cancer			0.0374	0.5754
No	676 (96.6)	671 (95.9)		
Yes	24 (3.4)	29 (4.1)		
Dementia			0.0805	0.1646
No	667 (95.3)	654 (93.4)		
Yes	33 (4.7)	46 (6.6)		
COPD			0.0218	0.7598
No	650 (92.9)	646 (92.3)		
Yes	50 (7.1)	54 (7.7)		
Hepatitis			0.0217	0.7868
No	673 (96.1)	670 (95.7)		
Yes	27 (3.9)	30 (4.3)		
Treatment strategy				0.082
ORIF	510 (72.9)	544 (77.7)	0.1128	
HA	189 (27)	154 (22)	0.1165	
THA	1 (0.1)	2 (0.3)	0.0309	
Time to admission (h)	97.42 ± 317.35	90.07 ± 222.51	0.0268	0.6157
Time to operation (d)	4.38 ± 2.70	4.54 ± 2.90	0.0591	0.2688
Operation time (mins)	90.69 ± 33.37	91.41 ± 33.05	0.0217	0.6845
Infusion (mL)	1499.36 ± 340.34	1504.63 ± 374.00	0.0147	0.7827

^*∗*^Variables were not successfully matched.

**Table 7:** Multivariate results by Cox regression (*N* = 1400).

Exposure	PSM model	PSM-adjusted model
Neutrophil	1.03 (1.00, 1.05) 0.0476	1.03 (1.00, 1.06) 0.0361
Neutrophil tertiles (^*∗*^10^9^/L)		
PSM low group (1.20–5.38)	1	1
PSM middle group (5.40–7.70)	1.04 (0.86, 1.25) 0.6886	1.05 (0.87, 1.27) 0.5957
PSM high group (7.71–24.29)	1.20 (1.00, 1.44) 0.0492	1.20 (1.00, 1.44) 0.0505
*P* for trend	0.0482	0.0498

Data in table: HR (95% CI) *P* value. Outcome variable: mortality. Exposed variables: neutrophil. Adjust variables in PSM-adjusted model: age, aCCI.

## Data Availability

The data are provided by Xi'an Honghui Hospital. According to relevant regulations, the data cannot be shared, but could be requested from the corresponding authors.

## References

[1] Waddell J. P. (2010). *Fractures of the Proximal Femur: Improving Outcomes E-Book: Expert Consult*.

[2] Egol K. A., Leucht P. (2017). *Proximal Femur Fractures: An Evidence-Based Approach to Evaluation and Management*.

[3] Kirilova E., Johansson H., Kirilov N. (2020). Epidemiology of hip fractures in Bulgaria: development of a country-specific FRAX model. *Archives of Osteoporosis*.

[4] Kanis J. A., Oden A., McCloskey E. V., Johansson H., Wahl D. A., Cooper C. (2012). A systematic review of hip fracture incidence and probability of fracture worldwide. *Osteoporosis International*.

[5] Chang S.-M., Hou Z.-Y., Hu S.-J., Du S. C. (2020). Intertrochanteric femur fracture treatment in Asia: what we know and what the world can learn. *Orthopedic Clinics of North America*.

[6] Tu W. J., Zeng X., Liu Q. (2022). Aging tsunami coming: the main finding from China’s seventh national population census. *Aging Clinical and Experimental Research*.

[7] Hagino H., Furukawa K., Fujiwara S. (2009). Recent trends in the incidence and lifetime risk of hip fracture in Tottori, Japan. *Osteoporosis International*.

[8] Malafarina V., Reginster J.-Y., Cabrerizo S. (2018). Nutritional Status and Nutritional treatment are related to outcomes and mortality in older Adults with hip fracture. *Nutrients*.

[9] Hu F., Jiang C., Shen J., Tang P., Wang Y. (2012). Preoperative predictors for mortality following hip fracture surgery: a systematic review and meta-analysis. *Injury*.

[10] Tajeu G. S., Delzell E., Smith W. (2014). Death, Debility, and Destitution following hip fracture. *The Journals of Gerontology: Series A*.

[11] Johnell O., Kanis J. A. (2004). An estimate of the worldwide prevalence, mortality and disability associated with hip fracture. *Osteoporosis International*.

[12] Loggers S. A. I., Van Lieshout E. M. M., Joosse P., Verhofstad M. H., Willems H. C. (2020). Prognosis of nonoperative treatment in elderly patients with a hip fracture: a systematic review and meta-analysis. *Injury*.

[13] Neuman M. D., Fleisher L. A., Even-Shoshan O., Mi L., Silber J. H. (2010). Nonoperative care for hip fracture in the elderly: the influence of race, income, and comorbidities. *Medical Care*.

[14] Cram P., Yan L., Bohm E. (2017). Trends in operative and nonoperative hip fracture management 1990–2014: a Longitudinal analysis of Manitoba Administrative data. *Journal of the American Geriatrics Society*.

[15] Elffors I., Allander E., Kanis J. A. (1994). The variable incidence of hip fracture in Southern Europe: the MEDOS study. *Osteoporosis International*.

[16] Liew P. X., Kubes P. (2019). The Neutrophil’s role during health and disease. *Physiological Reviews*.

[17] Kolaczkowska E., Kubes P. (2013). Neutrophil recruitment and function in health and inflammation. *Nature Reviews Immunology*.

[18] Dalli J., Montero-Melendez T., Norling L. V. (2013). Heterogeneity in neutrophil microparticles reveals distinct proteome and functional properties. *Mol Cell Proteomics*.

[19] Soehnlein O., Lindbom L. (2010). Phagocyte partnership during the onset and resolution of inflammation. *Nature Reviews Immunology*.

[20] Robertson A. L., Holmes G. R., Bojarczuk A. N. (2014). A Zebrafish Compound screen reveals Modulation of neutrophil Reverse Migration as an anti-inflammatory mechanism. *Science Translational Medicine*.

[21] Wang J. (2018). Neutrophils in tissue injury and repair. *Cell and Tissue Research*.

[22] Wang Z., Tian S., Zhao K. (2020). Neutrophil to lymphocyte ratio and fracture severity in young and middle-aged patients with tibial plateau fractures. *International Orthopaedics*.

[23] Forget P., Dillien P., Engel H., Cornu O., De Kock M., Yombi J. C. (2016). Use of the neutrophil-to-lymphocyte ratio as a component of a score to predict postoperative mortality after surgery for hip fracture in elderly subjects. *BMC Research Notes*.

[24] Chen Y. H., Chou C. H., Su H. H. (2021). Correlation between neutrophil-to-lymphocyte ratio and postoperative mortality in elderly patients with hip fracture: a meta-analysis. *Journal of Orthopaedic Surgery and Research*.

[25] Temiz A., Ersözlü S. (2019). Admission neutrophil-to-lymphocyte ratio and postoperative mortality in elderly patients with hip fracture. *Ulus Travma Acil Cerrahi Derg*.

[26] Bastian O. W., Koenderman L., Alblas J., Leenen L. P., Blokhuis T. J. (2016). Neutrophils contribute to fracture healing by synthesizing fibronectin + extracellular matrix rapidly after injury. *Clinical Immunology*.

[27] Kaiser K., Prystaz K., Vikman A. (2018). Pharmacological inhibition of IL-6trans-signaling improves compromised fracture healing after severe trauma. *Naunyn-Schmiedeberg’s Archives of Pharmacology*.

[28] Kovtun A., Bergdolt S., Wiegner R., Radermacher P., Huber-Lang M., Ignatius A. (2016). The crucial role of neutrophil granulocytes in bone fracture healing. *European Cells and Materials*.

[29] Fisher A., Srikusalanukul W., Fisher L., Smith P. (2016). The neutrophil to lymphocyte ratio on admission and short-term outcomes in Orthogeriatric patients. *International Journal of Medical Sciences*.

[30] Fischer V., Haffner-Luntzer M. (2022). Interaction between bone and immune cells: implications for postmenopausal osteoporosis. *Seminars in Cell & Developmental Biology*.

[31] Jaillon S., Galdiero M. R., Del Prete D., Cassatella M. A., Garlanda C., Mantovani A. (2013). Neutrophils in innate and adaptive immunity. *Seminars in Immunopathology*.

[32] Martínez de Toda I., Vida C., Díaz-Del Cerro E., De la Fuente M. (2021). The immunity Clock. *J Gerontol A Biol Sci Med Sci*.

[33] Dorshkind K., Montecino-Rodriguez E., Signer R. A. J. (2009). The ageing immune system: is it ever too old to become young again. *Nature Reviews Immunology*.

[34] García-Alvarez F., González P., Navarro-Zorraquino M. (2008). Immune cell variations in patients with hip fracture. *Archives of Gerontology and Geriatrics*.

[35] Fulop T., Le Page A., Fortin C., Witkowski J. M., Dupuis G., Larbi A. (2014). Cellular signaling in the aging immune system. *Current Opinion in Immunology*.

[36] Fortin C. F., McDonald P. P., Lesur O., Fulop T. (2008). Aging and neutrophils: there is Still Much to Do. *Rejuvenation Research*.

[37] Solana R., Tarazona R., Gayoso I., Lesur O., Dupuis G., Fulop T. (2012). Innate immunosenescence: effect of aging on cells and receptors of the innate immune system in humans. *Seminars in Immunology*.

[38] Baëhl S., Garneau H., Le Page A. (2015). Altered neutrophil functions in elderly patients during a 6-monthfollow-up period after a hip fracture. *Experimental Gerontology*.

[39] Auais M., Morin S., Nadeau L., Finch L., Mayo N. (2013). Changes in frailty-related characteristics of the hip fracture population and their implications for healthcare services: evidence from Quebec, Canada. *Osteoporosis International*.

[40] Bentler S. E., Liu L., Obrizan M. (2009). The Aftermath of hip fracture: discharge Placement, functional Status change, and mortality. *American Journal of Epidemiology*.

[41] Norring-Agerskov D., Bathum L., Pedersen O. B. (2019). Biochemical markers of inflammation are associated with increased mortality in hip fracture patients: the Bispebjerg Hip Fracture Biobank. *Aging Clinical and Experimental Research*.

[42] Bermejo-Bescos P., Martin-Aragon S., Cruz-Jentoft A. J., Merello de Miguel A., Vaquero-Pinto M. N., Sánchez-Castellano C. (2020). Peripheral IL-6 levels but not Sarcopenia are predictive of 1-year mortality after hip fracture in older patients. *Journals of Gerontology Series A: Biological Sciences & Medical Sciences*.

[43] de Miguel Artal M., Roca Chacon O., Martinez-Alonso M., Serrano Godoy M., Mas Atance J., Garcia Gutierrez R. (2018). Hip fracture in the elderly patient: prognostic factors for mortality and functional recovery at one year. *Revista Espanola de Geriatria y Gerontologia*.

[44] Barnes P. J. (2019). Inflammatory endotypes in COPD. *Allergy*.

[45] Karauda T., Kornicki K., Jarri A. (2021). Eosinopenia and neutrophil-to-lymphocyte count ratio as prognostic factors in exacerbation of COPD. *Scientific Reports*.

[46] Ha S. Y., Choi S., Park S. (2020). Prognostic effect of preoperative neutrophil-lymphocyte ratio is related with tumor necrosis and tumor-infiltrating lymphocytes in hepatocellular carcinoma. *Virchows Archiv*.

[47] Yoon C. I., Park S., Cha Y. J. (2020). Associations between absolute neutrophil count and lymphocyte-predominant breast cancer. *The Breast*.

